# Points from Hospital Reports

**Published:** 1924-10

**Authors:** 


### POINTS FROM HOSPITAL REPORTS.

### Bolingbroke Hospital.

The year 1923 was not only a very busy one—the number of patients, both in and out being greater than in 1922—but it was distinguished by an event which must become an important landmark in the history of the Hospital. In the Report of the preceding year the extension of the Hospital was foreshadowed, and the announcement was accompanied by the statement that the Trustees of the estate of the late Mr. William Shepherd had made a generous contribution of £10,000 for this purpose. The Governors now announce that the Trustees have promised a further magnificent donation for the extension, on the scheme being approved, of £25,000 by them. The King's Fund has promised a further £1,000, and as the total cost is put at £50,000, only £14,000 remains to be provided. We congratulate the Governors on their good fortune, and wish them speedy success in their efforts to raise the balance. The architects are Messrs. Young and Hall, and a perspective of the new building is given on page 2 of the Report. There were material reductions in the expenditure in 1923, greatly improving the averages; and although, largely owing to reductions in the charges to private patients, the income was £1,258 less than in the preceding year, it was still more than sufficient to meet the total expenditure. There appears to be no index to the Report. We hope to see the want supplied in the next issue.

### Royal London Ophthalmic Hospital (Moorfields Eye Hospital).

This, one of the most valuable and justly renowned of the Hospitals of London, had a very busy year in 1923. Its available beds were kept fully occupied throughout the year, and the number of out-patients was greatly increased. It is sad to see that 18 of its 138 beds were closed throughout 1923 because there was not sufficient nursing accommodation to house an adequate staff, and money is not available for the necessary building and equipment to provide them. The wide dissemination of this tragic fact should bring a speedy remedy. That the Moorfields Eye Hospital should be seriously hampered in its magnificent work because it cannot obtain the money to expand its Nurses' Home to the necessary dimensions is almost incredible. But it has come to pass, and the sooner the conscience of the whole country is quickened about it the better. It should not be necessary to say that the Hospital is a national Institution, or, having said it, to point the moral.

### The New Sussex Hospital for Women and Children, Brighton.

This hospital of 30 beds was opened in 1912 for patients desiring to be treated by women doctors, who are unwilling to go into the free wards of a public hospital but are unable to meet the fees of nursing homes. Out-patients are attended free, but a charge of one shilling is made for medicines, &c. In 1921 the hospital was moved to its present building on a beautiful site of two acres. The cost of the land and building and of the conversion and fitting up of the latter as a hospital was £13,576, the whole of which has been provided. There are no statistical tables in the report which would enable us to compare 1923 with past years in regard to work, income and expenditure. We learn, however, from the Report of the Board of Management that the number of in-patients last year was 392, that out-patients numbered 1,945—300 more than in 1922, that there was a slight increase in expenditure, which amounted to £4,907, and that the income was sufficient to meet it and leave a small balance. It appears that there are only three hospitals in the country, of which this is one, staffed entirely by women doctors. The Report would be improved by an index and some statistical tables.

### Carnarvonshire and Anglesey Infirmary, Bangor.

The great event of the year 1923, and, perhaps, the greatest in the history of the Infirmary, was the laying of the foundation stone of the New Wing by the Prince of Wales. It appears that the new building is expected to cost £20,000. Of this £19,000 has been raised, and a special effort is now being made to obtain the last £1,000—always the most difficult to find. A friend of the Hospital has promised £50 on condition that nineteen others give a like sum. This should not, and we are confident will not prove to be beyond the powers of the people of two important Welsh Counties. But there are many men—and women—of Carnarvon and Anglesey, who have sought—and found—their fortunes far from their native place, and if this paragraph should catch the eye of any of them we doubt not that their obvious duty will meet with immediate and cheerful practical recognition. We think the Report might contain information as to the number of beds in the Hospital, the average number in occupation throughout the year, the number of out-patients, and other customary particulars. Some facts about the accommodation provided in the New wing would also be very interesting, and when interest is excited, sympathy is near at hand, and practica help but a step further away.

### Beckett Hospital and Dispensary, Barnsley.

This Hospital of 120 beds worked at high pressure during 1923. There was a remarkable increase, from 88 to 105, in the average number of in-patients resident daily, and a considerable one in the number of out-patients. This was, of course, reflected in the expenditure, but that the latter is well controlled is shown by the fact that the average cost per head per week was less than in 1922 by 3s. 4d. There was, however, a very slight increase in the average cost per out-patient (which, no doubt by a printer's mistake is put at £4 8s. 3d. instead of 4s. 8·3d.), but a reduction in the average cost per attendance of about 1¼d. Although the income was £338 in excess of 1922, it was insufficient to meet the expenditure by £1,458, a fact which causes the Committee an anxiety which they hope will be relieved next year. Extensions of the Hospital, to cost £34,000, are under consideration, and it is the intention of the Committee to proceed at once with an appeal for the first section of the work, which is to cost £10,000. There is on page 51, without a heading, a puzzling array of figures of Income and Expenditure from 1881 to 1923. We have tried unavailingly to discover to what these figures relate. Obviously not to the Hospital itself. We think an index to the Report would be an improvement.

### The Infants' Hospital, Vincent Square, Westminster.

The Infants' Hospital, 50 beds, was incorporated during the year under review—1923. Alterations and improvements, and repairs and decorations accumulated since 1914, were carried out during the year, and necessarily forced up the expenditure above that of 1922. There were great increases, too, in the number both of in-patients and out-patients. The average cost of patients was very high, but the inflated expenditure of which we have spoken, together with the fact that not all the cots in the hospital are in use, of course militates against good averages of cost. All this but goes to emphasise the fact that one essential element of economical administration is a full hospital. We are not animadverting on the management of the Infants' Hospital—it appears not to be practicable at present for all the cots to be in use—but are simply pointing out that normal average costs are attainable only if the hospital is maintained regularly in full operation. Here, last year, only 25 of the 50 beds were in regular occupation.

### Bombay Mental Hospitals.

The Annual Report for 1922 on the six mental hospitals in the Bombay Presidency presents some interesting details. The total population was 1,905 as compared with 1,938 in the previous year. There were 454 admissions against 501 in 1921. Of the total treated 267 were "cured"—it would be interesting to know here as elsewhere what exactly constitutes a "cure" in cases of mental disorder—and 95 were transferred to the care of friends. Of the latter 62 had improved while under treatment. During the year the daily average of sick was 58.0 as compared with 61.4 in the previous year. The general health of the insane was satisfactory at all the mental hospitals. The ratio of deaths to the daily average strength was 8.2 against 10.9. The chief causes of death were tuberculosis 17, diarrhœa 14, anæmia 9, diseases of the heart 13, dysentery 10, and pneumonia 11. The patients were natives, except for 36 Europeans, Eurasians and other races. It is accordingly worthy of note that in 131 cases the cause of the insanity was "Cannabis Indica" or its preparations, while 63 were due to the "abuse of intoxicants."

